# Maresin 1 Alleviates Diabetic Kidney Disease via LGR6-Mediated cAMP-SOD2-ROS Pathway

**DOI:** 10.1155/2022/7177889

**Published:** 2022-04-19

**Authors:** Xinyue Li, Butuo Xu, Jing Wu, Yueli Pu, Shengrong Wan, Yan Zeng, Mei Wang, Lifang Luo, Fanjie Zhang, Zongzhe Jiang, Yong Xu

**Affiliations:** ^1^Department of Endocrinology and Metabolism, The Affiliated Hospital of Southwest Medical University, Luzhou, Sichuan 646000, China; ^2^Sichuan Clinical Research Center for Nephropathy, Luzhou, Sichuan 646000, China; ^3^Metabolic Vascular Disease Key Laboratory of Sichuan Province, Sichuan 646000, China; ^4^Department of Endocrinology and Metabolism, The Affiliated Traditional Chinese Medicine Hospital of Southwest Medical University, Luzhou, Sichuan 646000, China

## Abstract

Background. Chronic hyperglycemia-induced inflammation is recognized as the most important pathophysiological process in diabetic kidney disease (DKD). As maresin 1 (MaR1) is an extensive anti-inflammatory lipid mediator, the present study investigated the protective role of MaR1 in the pathogenesis of DKD and its clinical relevance. Methods. Serum MaR1 concentrations were analyzed in 104 subjects with normal glucose tolerant, type 2 diabetes (T2DM), or DKD. Streptozotocin (STZ) together with high fat diet was used to induce male C57BL/6 J mice into diabetic mice which were treated with MaR1. Human renal tubule epithelial cells (HK-2 cells) were treated by high glucose for glucotoxicity cell model and transfected with LGR6 siRNA for knockdown with MaR1 added，and detected oxidative stress and inflammatory related factors. Results. Serum MaR1 concentrations were significant decreased in T2DM with or without kidney disease compared with normal participant and were lowest in patients with DKD. Serum MaR1 concentrations were negatively correlated with hemoglobin A1c (HbA1c), duration of diabetes, urinary albumin to creatinine ratio (UACR), neutrophil, and neutrophil-lymphocyte ratio and were positively correlated with high-density lipoprotein-cholesterol (HDL-C) and estimated glomerular filtration rate (eGFR). In mouse model, MaR1 injection alleviated hyperglycemia, UACR and the pathological progression of DKD. Interestingly, the renal expression of LGR6 was down-regulated in DKD and high glucose treated HK-2 cells but up-regulated by MaR1 treatment. Mechanistically, MaR1 alleviated inflammation via LGR6-mediated cAMP-SOD2 antioxidant pathway in DKD mice and high glucose treated HK-2 cells. Conclusions. Our study demonstrates that decreased serum MaR1 levels were correlated with the development of DKD. MaR1 could alleviate DKD and glucotoxicity-induced inflammation via LGR6-mediated cAMP-SOD2 antioxidant pathway. Thus, our present findings identify MaR1 as a predictor and a potential therapeutic target for DKD.

## 1. Introduction

Diabetic kidney disease (DKD) is the most common diabetic microvascular complication and the main cause of end-stage renal disease worldwide [1]. However, the etiology and pathogenesis of DKD are complex and not completely clear at present. There is no specific treatment for DKD and its prognosis is usually poor. Therefore, it is essential to further explore the pathophysiological mechanism of DKD and clinical treatment strategies.

Hyperglycaemia-induced vascular dysfunction is the primary initiating mechanism in DKD, but its progression is also driven by a heterogeneous set of pathological mechanisms, including oxidative stress, inflammation and fibrosis [2]. Epithelial mesenchymal transition (EMT) and endothelial mesenchymal transition (EndMT) programs play vital roles in the development of fibrosis in the kidney [3]. EndMT is regulated by a combination of various inflammatory cytokine pressures, such as IL-1*β*, transforming growth factor-beta (TGF-*β*) [4]. Moreover, hyperglycaemia stimulates excess Reactive oxygen species (ROS) production, causing cell death, fibrosis and eventual kidney dysfunction [5]. Individually, the theories proposed thus far may not be able to explain the progression of DKD. Many signaling pathways have been proved to be involved in the occurrence and development of DKD. TGF-*β*/Smad and Wnt/*β*-catenin pathways are found to promote renal fibrosis concertedly or independently [6]. Recent studies report that endothelial glucocorticoid receptor or podocyte-specific glucocorticoid receptor loss is one of the catalysts of renal fibrosis in diabetes by up regulating canonical Wnt signaling [7, 8]. Moreover, Notch signaling pathway, hedgehog interacting protein, dipeptidyl peptidase-4 (DPP-4), decreasing sirtuin3 (SIRT3) and endothelial Fibroblast Growth Factor Receptor 1 (FGFR1) deficiency also result in renal fibrogenesis in DKD [9–13]. These pathways influence and link with each other and jointly act on the occurrence and development of DKD. The use of renin-angiotensin system (RAS) blocking agents (angiotensin-converting enzyme inhibitors (ACEi) and angiotensin 2 receptor blockers (ARB)) to control hyperglycemia combined with albuminuria reduction has been a cornerstone of nephropathy prevention and treatment for the past two decades [14]. It is worth mentioning that the study has shown that ACEi elevate N-acetyl-seryl-aspartyl-lysyl-proline (AcSDKP) level which contributes to anti renal fibrosis whereas ARB does not [15]. Recent evidence supports a renoprotective effect of sodium-glucose cotransporter 2 (SGLT2) inhibitions and glucagon-like peptide 1 (GLP-1) receptor agonists in addition to their hypoglycemic properties [14, 16, 17], where dapagliflozin and empagliflozin are most effective for kidney function progression [18, 19]. In multiple clinical trials, DPP-4 inhibitor linagliptin has been reported to reduce albuminuria in DKD, but neutral outcomes for the secondary renal endpoint (end stage renal disease, renal death or 40% reduction in eGFR) is reported [20–23]. In addition, study shows adding mineralocorticoid antagonist to existing RAS inhibitor therapy in patients with type 2 diabetes (T2DM) and microalbuminuria reduce progression of albuminuria to higher levels [24, 25]. Moreover, the beneficial effects of AcSDKP, glycolysis inhibitors, SIRT3 and ROCK inhibition also have been described in animal models of DKD [13, 26–28]. In conclusion, the mechanisms and pathways of DKD are complex, but obviously inflammation-related molecules and pathways are critically involved in the progression of DKD [29]. At present, there are few clinical drugs for DKD, and the prognosis is poor.

Maresin 1 (MaR1) synthesized by macrophages from docosahexaenoic acid (DHA) have anti-inflammatory and pro-resolving capacities as well as tissue regenerating and pain-relieving properties [30]. MaR1 has been reported to have benefits on multiple diseases, such as lung diseases, liver disease, vascular disease, sepsis and so on through its contributing roles to the regression of inflammation [31]. Yoshifumi Morita et al. have reported that urinary MaR1 were lower in the subjects with stage 3–4 diabetic nephropathy than the subjects with stage 1 or 2 diabetic nephropathy and in the control group [32]. Research on mouse glomerular mesangial cells (GMCs) has reported that MaR1 protect GMCs from glucotoxicity by mitigating ROS and the inflammation [33]. However, the mechanism of MaR1 in mitigating ROS and its role in the occurrence and development of DKD have not been fully clarified.

Two distinct types of receptor molecules have been reported as the targets of MaR1, including leucine-rich repeat domain-containing G protein-coupled receptor 6 (LGR6) and retinoic acid-related orphan receptor *α* (ROR*α*) [34]. It has been reported that cyclic adenosine 3′,5′-monophosphate (cAMP), a second messenger following G protein-coupled receptor (GPCR) activation, has been determined to be evoked by MaR1 with activating LGR6 [35]. Norihiro Sugino et al. demonstrated Mn-SOD (SOD2) activity was significantly increased by cAMP [36]. SOD2-mediated pathways have been shown to weaken the deleterious effect of ROS during hepatic ischemia-reperfusion injury (IRI) [37]. However, it is not clear whether MaR1 can activate cAMP -SOD2 through LGR6, thus inhibiting ROS in DKD.

The present study shows a correlation between serum MaR1 levels and DKD progression in human patients, and the effect of MaR1 on DKD mice was further explored through animal experiments. The last, we explored the mechanism of alleviation of DKD through MaR1 via LGR6-mediated cAMP-SOD2 antioxidant pathway in vitro. Taken together, our findings, involving in-vitro and in-vivo studies at cell, animal and human levels, suggest MaR1 as a promising new therapy for DKD via targeting LGR6-mediated antioxidant pathway.

## 2. Materials and Methods

### 2.1. Human Serum, Urine, and Renal Biopsy Samples

A total of 60 T2DM patients recruited from the Department of Endocrinology and Metabolism, Affiliated Hospital of Southwest Medical University from March to June 2021 were selected for observation, including 36 T2DM patients (without kidney disease) and 24 DKD patients. In addition, 44 healthy subjects were recruited for comparison from the Health Examination Center. The inclusion criteria were as follows: (1) age>18 years old, (2) the T2DM diagnosed according to the 1999 World Health Organization (WHO) [38], (3) DKD diagnosed according to the American Diabetes Association (ADA) guideline [39]. The exclusion criteria were as follows: (1) pregnant and lactating women, (2) acute complications of diabetes, (3) malignant tumor, (4) various non-diabetic kidney diseases, (5) all kinds of acute and chronic infectious diseases and blood system diseases that may lead to abnormal blood routine, (6) thyroid dysfunction, (7) gout, and (8) use of antibiotics or hormone. Subjects in the study had no disease that affect the inflammatory system. All fasting blood and urine samples were separated within 2 h of collection, centrifuged 20 minutes (3000 revolutions per minute, 4°C), and then frozen at –80°C until needed for the study.

Renal biopsy specimens from T2DM patients (n =3) with pathological diagnosis of DKD were obtained from Department of Pathology, the Affiliated Hospital of Southwest Medical University. The control samples (n =3) were taken from healthy kidney poles of individuals who underwent cancer nephrectomy without other renal diseases.

All experimental protocols were registered online (Clinical trial register no. ChiCTR2100048381) and approved by the Ethics Committee of The Affiliated Hospital of Southwest Medical University. Our study was carried out in accordance with the Helsinki Declaration. Written, informed consent was obtained from our patients in the study.

### 2.2. Animals

A total of 17 six-week-old male C57BL/6 J mice (GemPharmatech Co., Ltd, Nanjing, China) were used in this study. The mice were randomly divided into the following experimental groups: normal control mice (normal group, n =5), DKD mice induced by high-fat diet (HFD, 60% fat) combined with continuous intraperitoneal injection of low-dose streptozotocin (STZ, Solarbio Science & Technology Co., Beijing, China) (DKD group, n =7), and MaR1-treated DKD mice (DKD + MaR1 group, n =5). DKD model was induced by HFD and STZ through intraperitoneal injection at a dose of 50 mg/kg for seven consecutive days. Mice with fast blood glucose levels over 16.7 mM were considered diabetes [40–42]. MaR1 was used to treat the mice at a dose of 4 ug/kg via intraperitoneal injection after STZ injection with 24-hour intervals for 14 weeks. The mice were kept on an alternating 12 h light-dark cycle at 23 ± 1°C in individually ventilated cages, with 60 ± 10% relative humidity. All mice were killed after 14 weeks of treatment. During the experiments, body weights and blood glucose concentrations were measured every 2 weeks. Animal experiments were approved by the Institutional Animals Ethics Committees of Southwest Medical University and in accordance with the National Institutes of Health (NIH) guidelines for the care and use of laboratory animals.

### 2.3. Cell Culturation

Human renal proximal tubular epithelial cells (HK-2) (ATCC, USA) were cultured in DMEM/F12 (Invitrogen, Carlsbad, CA, USA) with 10% fetal bovine serum (FBS, Sciencell, USA) and 1% penicillin/streptomycin (PS, Beyotime, Shanghai, China) at 37°C and 5% CO2 [43]. MaR1 (100 *μ*M, Cayman Chemical, MI, USA) was added to high glucose (HG) for 72 h to investigate the effects on high glucose-induced HK-2 cells.

### 2.4. Clinical Evaluation of Subjects

General data including gender, age, and duration of diabetes of all subjects were collected. The height and weight were measured by the same staff member, and the body mass index (BMI) was calculated, according to the formula: BMI = weight/height^2^ (kg/m^2^). After the participants had rested for 10 min, the systolic blood pressure (SBP) and diastolic blood pressure (DBP) of both upper limbs were measured by the same professional staff twice and the average value was recorded as the final blood pressure value.

Overnight urine and fasting blood samples were collected for determinations of urine albumin to creatinine ratio (UACR), hemoglobin A1c (HbA1c), triglycerides (TG), total cholesterol (TC), high-density lipoprotein cholesterol (HDL-C), low-density lipoprotein cholesterol (LDL-C), Uric Acid (UA), creatinine, estimated glomerular filtration rate (eGFR), neutrophil and lymphocyte. UACR was measured by turbidimetry immunoassay and enzyme colorimetry. HbA1c was tested by high performance liquid chromatography (HPLC). TG, TC, HDL-C, LDL-C, UA, creatinine and eGFR were determined using enzymatic method. Neutrophil and lymphocyte were determined using flow cytometry.

### 2.5. Assessment of Human Serum MaR1 Levels, cAMP Levels, Inflammatory Cytokines and Animal Urinary Protein

Human serum MaR1 levels were measured in the extracted serum using commercial competitive ELISA kits (Cayman Chemical, MI, USA). The cAMP content in HK-2 cells and renal tissue homogenate were measured by cAMP ELISA kits (Cell Signaling Technology, USA). The IL-18 and IL-1*β* content of HK-2 cells supernatant were measured using human IL-18 ELISA kits (Andygene, China), and human IL-1*β* ELISA kits (Andygene, China). Urinary protein and creatinine of mice was measured using commercial determination kits (Nanjing Jiancheng Bioengineering Institute, China). The above substances were detected by ELISA method according to the manufacturer's instructions.

### 2.6. Histopathological Examination

Histopathological examination was as described by our previous study [44]. Kidney tissues were fixed in 4% paraformaldehyde (PFA), embedded in paraffin and cut into 4-*μ*m-thick sections for histological analysis. Sections were stained with hematoxylin-eosin (HE) for morphometry.

### 2.7. Immunohistochemistry Staining

Kidney sections of 4 *μ*m were dewaxing hydration and stained with primary antibodies against LGR6 (1 : 100, SC-393010, Santa Cruz), kidney injury molecule-1 (KIM-1)(1 : 100, ab78494, abcom), SOD2 (1 : 100, 13141 T, Cell Signaling Technology), IL-18 (1 : 100, AF5207, Beyotime), and IL-1*β* (1 : 100, AF7209, Beyotime). The sections were stained with biotin-labeled goat anti-rabbit IgG or biotin-labeled anti-mouse IgG and then treated with the Horseradish enzyme labeled oopaltin of Streptomyces (Beijing ZSGB Biological Technology CO., LTD. China). The photograph of each stained sections was captured by the light microscope.

### 2.8. Immunofluorescence Staining

Immunofluorescence (IF) staining for kidney paraffin sections were stained with LGR6 antibody (1 : 100, SC-393010, Santa Cruz). Cy3/FITC fluorescent dye-conjugated secondary antibody (1 : 200, Biosynthesis Biotech, China) were incubated in the dark for 60 min. Nucleus was labeled with DAPI, and images were obtained using a fluorescence microscope (Leica, Germany).

### 2.9. Western Blotting Analysis

Proteins of HK-2 cells or mice kidney tissues were extracted with extraction buffer (RIPA) (Beyotime, Shanghai, China) added with 1% protease inhibitor (Beyotime, Shanghai, China). Samples were separated by sodium dodecyl sulfate-polyacrylamide gel electrophoresis (SDS-PAGE) and subsequently transferred into PVDF membranes (Millipore, MA, USA). The membranes were blocked with 5% BSA and then incubated with anti-LGR6 antibody (1 : 1000, ab126747, Abcam,), anti-SOD2 antibody (1 : 1000, 13141 T, Cell Signaling Technology), anti-IL-18 antibody (1 : 1000, AF5207, Beyotime), anti-IL-1*β* antibody (1 : 1000, AF7209, Beyotime). Then, the membranes were incubated with the corresponding secondary antibody (HRP-tagged goat anti-mouse or anti-rabbit IgG) and detected by enhanced chemiluminescence reagents (ECL, Millipore, USA). Quantitative analysis was performed with the Image J software.

### 2.10. Measurement of ROS

ROS content was measured with DCFH-DA fluorescent probe for 20 min at 37°C after drug intervention according to the ROS Assay Kit protocol (Beyotime, China).

### 2.11. siRNA Transfection

According to the instructions, the LGR6 siRNA and control siRNA were transfected into HK-2 cells with riboFECTTM CP Reagent and riboFECTTM CP Buffer (RiboBio, Guangzhou, China) at 100 nM of siRNA. After siRNA transfection, the cells were treated with HG or MaR1 for subsequent experiments. The siRNA sequence of human LGR6 gene was: 5'-TCCAGTACCTGCCTAAACT-3'.

### 2.12. qRT-PCR Analysis

Total RNAs of renal tissue and HK-2 cells were extracted with the Trizol (Invitrogen). The ReverTra Ace qPCR RT Master Mix (FSQ-201, TOYOBO) was used for reverse transcription reaction and QuantiNova SYBR Green PCR Kit (QIAGEN, German) was used for qRT-PCR. The qRT-PCR was performed with Analytikjena qTOWER 3G real-time PCR system (JENA, German) according to the manufacturer's instructions. The primers used in this study were given in Supplemental Table 1. We selected GAPDH as the endogenous control. All the samples were used in triplicates. The 2^-△△Ct^ method [45] was used to calculate the relative gene expression compared with the reference gene.

### 2.13. Statistical Analyses

The SPSS 22.0 software was used to perform all statistical analyses. The normality of continuous variables was assessed with Shapiro-Wilk's test. Data were expressed as the mean ± SD for normally distributed values or the median (25-75th percentiles) for nonparametric values, and categorical variables were expressed as ratios. Difference between groups was analyzed using one-way ANOVA for normally distributed values and Kruskal–Wallis H test for nonparametric values, or the chi-squared test for categorical variables. Interrelationships between variables were estimated using Spearman's correlation coefficient with or without adjusting for age and sex. Multivariate logistic regression analyses were used to analyze the association between serum MaR1 concentrations and T2DM without or with DKD. All reported *P* values were two tailed. Differences were evaluated using P <0.05 was considered statistically significant.

## 3. Results

### 3.1. Serum MaR1 Concentrations Were Lower in Patients with DKD than That in Diabetes without Kidney Disease

**Figure 1:**
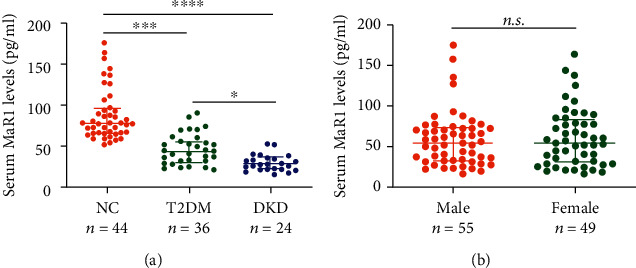
MaR1 levels in different groups. (a) Circulating MaR1 levels in normal, T2DM and DKD groups. (b) Circulating MaR1 levels in subgroups stratified by gender. Each data point represents a serum sample, the horizontal middle line in each data set represents the median, and the limits of the vertical lines represent the interquartile range. One-way ANOVA with Tukey's post hoc test was performed for multiple comparisons. ∗∗∗∗*P* < 0.0001; ∗∗∗*P* < 0.001; ∗*P* < 0.05; n.s.: not significant.

To investigate the role of MaR1 in the pathogenesis of DKD, we included 44 normal and 60 diabetic participants (36 diabetes without kidney disease and 24 diabetes with kidney disease) to detect the serum levels of MaR1. The comparative demographic and laboratory parameters of the included participants were given in Supplemental Table 2. Notably, we found that serum MaR1 concentrations were significantly decreased in T2DM patients whether with or without kidney disease compared with normal subjects. Moreover, serum MaR1 concentrations were lower in patients with diabetic kidney disease than that in diabetes without kidney disease (Figure 1(a)). Besides, when all participants were stratified by gender, there were no significant differences in serum MaR1 concentrations between men and women (Figure 1(b)). These data indicate that the reduction of MaR1 levels is clinically associated with the pathogenesis of DKD.

### 3.2. Serum MaR1 Concentrations Were Negatively Correlated with UACR, HbA1c, Duration of Diabetes, Neutrophil, Neutrophil to Lymphocyte Ratio (NLR) and Positively Associated with HDL-C, eGFR in Participants

**Figure 2:**
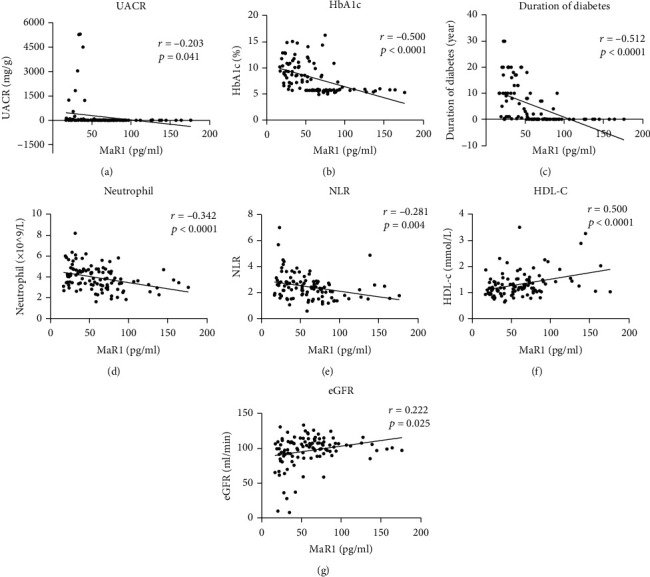
Correlation between circulating MaR1 levels and UACR (a), HbA1c (b), duration of diabetes (**c**), neutrophil (d), NLR (e), HDL-C (f), eGFR (g). UACR: urine albumin to creatinine ratio; HbA1c: hemoglobin A1c; NLR: neutrophil to lymphocyte ratio; HDL-C: high-density lipoprotein-cholesterol; eGFR: estimated glomerular filtration rate.

Next, we investigated the association of circulating MaR1 concentrations and metabolic-related parameters. Serum MaR1 concentrations were negatively correlated with UACR, SBP, HbA1c, duration of diabetes, neutrophil, and NLR (all *P* <0.05, Supplemental Table 3) and were positively correlated with HDL-C, eGFR (all *P* <0.05, Supplemental Table 3). After adjusting for age and sex, MaR1 remained statistically negatively correlated with UACR, HbA1c, duration of diabetes, neutrophil and NLR (all *P* <0.05, Figure 2(a)–2(e)) and positively associated with HDL-C, eGFR (all *P* <0.05, Figures 2(f) and 2(g)). Taken together, these findings suggest a strong negative correlation between MaR1 concentrations and the pathogenesis of DKD.

Multivariate logistic regression analysis also reveal that decreased serum MaR1 concentrations are significantly associated with T2DM without or with DKD after controlling for age, sex, BMI, blood pressure, and lipid profiles (odds ratio, 0.889 and 0.758, 95% confidence interval 0.840-0.942 and 0.676-0.850, both *P* <0.0001) (Supplemental Tables 4a and 4b).

### 3.3. MaR1 Ameliorates Hyperglycemia and Renal Dysfunction in DKD Mouse Model Induced by HFD Combined with STZ

**Figure 3:**
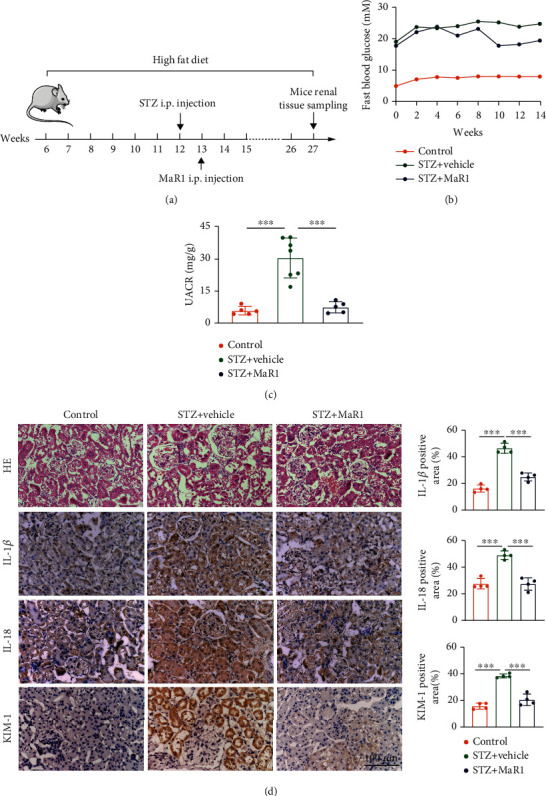
MaR1 ameliorated hyperglycemia and renal dysfunction in DKD mouse model. (a) Experimental design for MaR1 treatment DKD mice induced by HFD combined with STZ. (b) Mean blood glucose level of mice in indicated group (n =5 in control group, n =7 in DKD group and n =5 in DKD+ MaR1 group). (c) Urinary albumin to creatinine ratio (UACR) of mice in indicated group (n =5 in control group, n =7 in DKD group and n =5 in DKD + MaR1 group). (d) The representative photomicrographs of HE and immunohistochemistry (IL-1*β*, IL-18 and KIM-1) showing the pathological changes in kidneys from indicated groups. Bar: 100 *μ*m. Data were expressed as mean ± SD; ∗∗∗P <0.001.

Based on the clinical relevance, we predicted that the loss of MaR1-mediated anti-inflammatory function led to the progression of DKD. To this end, we next explored the protective effect of MaR1 in DKD mouse model induced by HFD combined with STZ. The experimental strategy is shown in Figure 3(a). After MaR1 injection for 3 months, we detected the blood glucose and UACR, which are the most important function indicator of DKD. Notably, the MaR1 injection remarkably ameliorated the hyperglycemia of DKD mice (Figure 3(b)). Moreover, we also observed significantly lower UACR in MaR1-treated DKD mice than that in the vehicle-treated DKD mice (Figure 3(c)). In further histological analysis, H&E staining suggested that renal morphology of kidneys from MaR1-injected DKD mice showed normal morphology of glomerulus, unlike the glomerular hypertrophy in vehicle-injected mice (Figure 3(d)). The DKD pathology-related inflammation factors, IL-18 and IL-1*β*, were also substantially reduced after MaR1 injection (Figure 3(d)). In addition, immunostaining assay showed KIM-1 levels were increased in kidneys from DKD mice, which were reversed by MaR1 treatment (Figure 3(d)). However, the average body weight of DKD mice treated with MaR1 and vehicle treated DKD mice showed no significant difference (Supplemental Figure 1). These findings collectively provide strong support for the promising application of MaR1 as a therapeutic inflammatory regression mediator in DKD.

### 3.4. MaR1 Receptor LGR6 Was Reduced in DKD and Reversed by MaR1 Treatment

**Figure 4:**
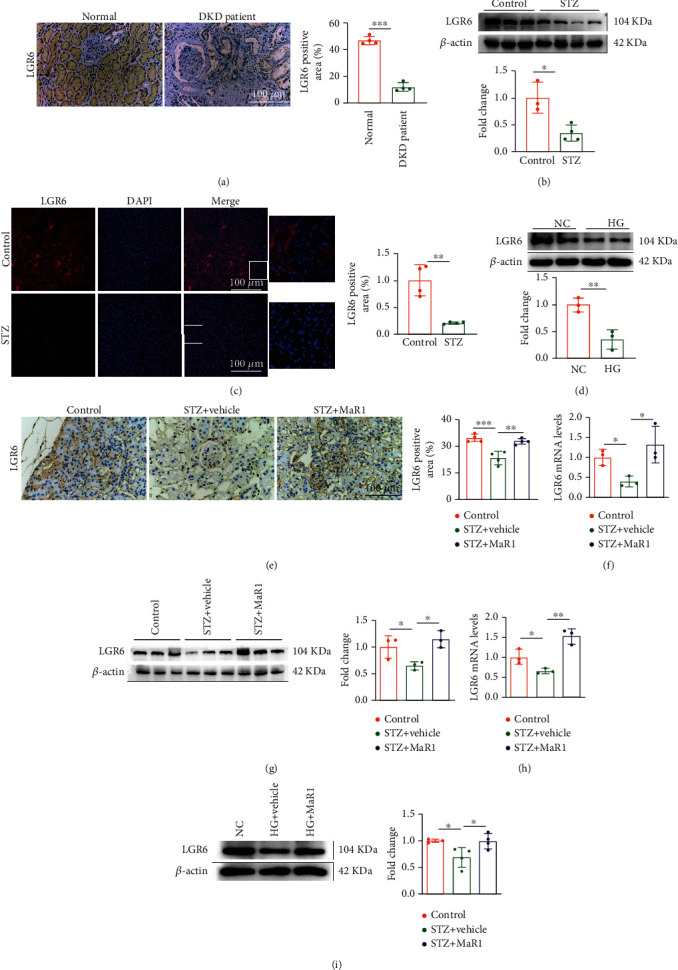
LGR6 was reduced in DKD and reversed by MaR1 treatment. (a) Representative immunohistochemistry images showing the localization and expression of LGR6 in kidney of DKD patients. Bar: 100 *μ*m. (b) Representative western blotting analysis images showing the protein levels of LGR6 in DKD mouse model. (c) Representative immunofluorescence images showing the localization and expression of LGR6 in kidney of mouse model. Bar: 100 *μ*m. (d) Representative western blotting analysis images showing the protein levels of LGR6 in HK-2 cells stimulated by high glucose (40 nM). (e) Representative immunohistochemistry images showing the localization and expression of LGR6 in kidney of DKD mouse model with MaR1 intervention. Bar: 100 *μ*m. (f) qRT-PCR showing the mRNA levels of LGR6 in DKD mouse model with MaR1 intervention. (g) Representative western blotting analysis images showing the protein levels of LGR6 in DKD mouse model with MaR1 intervention. (h) qRT-PCR showing the mRNA levels of LGR6 in high glucose-stimulated HK-2 cells with MaR1 intervention. (i) Representative western blotting analysis images showing the protein levels of LGR6 in high glucose-stimulated HK-2 cells with MaR1 intervention. All results are representative of three independent experiments. Data were expressed as mean ± SD; ∗P <0.05; ∗∗P <0.01; ∗∗∗P <0.001.

As LGR6 is one of the key receptors of MaR1 [34] and mediates protective effect in many diseases [46–48], we speculated that MaR1 might function through LGR6. To clarify this, we firstly detected the LGR6 expression levels in clinical samples from normal and DKD patients. In contract to normal participants, expression levels of LGR6 were remarkably reduced in kidneys from DKD patients (Figure 4(a)). Similarly, western blot and immunostaining assay, respectively, showed decreased expression levels of LGR6 in kidneys from DKD mouse model (Figures 4(b) and 4(c)). Of note, immunostaining assay also showed that LGR6 was mainly expressed in renal tubular cells (Figure 4(c)). Moreover, the expression of KIM-1, a marker of renal tubular injury, was significantly enhanced in DKD mice compared with normal mice (Figure 3(d)). Thus, we used a renal tubular cell line, HK-2, to establish a glucotoxicity cell model. As shown in Figure 4(d), LGR6 was significantly reduced by high glucose treatment in HK-2 cells. Interestingly, MaR1 injection could reverse the reduction of LGR6 protein levels in kidneys from DKD mouse model, which was also consistent in mRNA levels (Figure 4(e)–4(g)). Consistent with that in mouse model, MaR1 treatment also reversed high glucose-induced LGR6 reduction in HK-2 cells (Figure 4(h)–4(i)). These data indicate that MaR1 might ameliorate DKD through LGR6.

### 3.5. MaR1 Reversed High Glucose-Induced ROS Overproduction through cAMP-SOD2 Antioxidant Pathway

**Figure 5:**
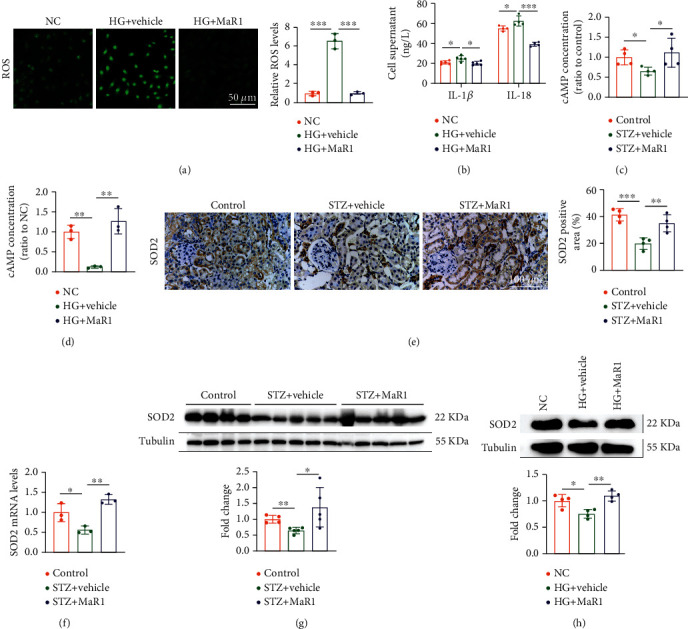
MaR1 reversed high glucose-induced ROS overproduction through cAMP-SOD2 antioxidant pathway. (a) DCFH-DA probe was used to detect the levels of ROS in high glucose-stimulated HK-2 cells with MaR1 intervention. Bar: 50 *μ*m. (b) ELISA assay showing the levels of IL-1*β* and IL-18 in cell culture supernatant of high glucose-stimulated HK-2 cells with MaR1 intervention. (c) The concentrations of cAMP in DKD mouse kidney with MaR1 intervention. (d) The concentrations of cAMP in high glucose-stimulated HK-2 cells with MaR1 intervention. (e) Representative immunohistochemistry images showing the protein levels of SOD2 in DKD mouse kidney with MaR1 intervention. (f) qRT-PCR showing the mRNA levels of SOD2 in DKD mouse kidney with MaR1 intervention. (g) Representative western blotting analysis images showing the protein levels of SOD2 in DKD mouse kidney with MaR1 intervention. (h) Representative western blotting analysis images showing the protein levels of SOD2 in high glucose-stimulated HK-2 cells with MaR1 intervention. All results are representative of three independent experiments. Data were expressed as mean ± SD; ∗P <0.05; ∗∗P <0.01; ∗∗∗P <0.001.

MaR1 have been reported to function in an LGR6-dependent manner by cAMP [35], which could increase the activity of SOD2 [36], a strong antioxidant enzyme. Here we also found that MaR1 significantly inhibited high glucose-induced ROS (Figure 5(a)) and the ROS-induced secretion of pro-inflammatory factors (Figure 5(b)). Thus, we next sought to determine whether MaR1 function as an antioxidant through cAMP/SOD2 mediated antioxidant pathway. As shown in Figures 5(c) and 5(d), cAMP levels were reduced both in kidneys from DKD mice and high glucose-treated HK-2 cells, which were obviously reversed by MaR1 treatment. Likewise, mRNA and protein levels of SOD2 were also decreased in kidneys from DKD mice and high glucose-treated HK2 cells, which were increased by MaR1 treatment (Figures 5(e)–5(h)). Taken together, these data suggest that MaR1 might ameliorate DKD through cAMP/SOD2 mediated antioxidant pathway.

### 3.6. MaR1 Alleviates High Glucose-Induced Inflammation via LGR6-Mediated Antioxidant Pathway

**Figure 6:**
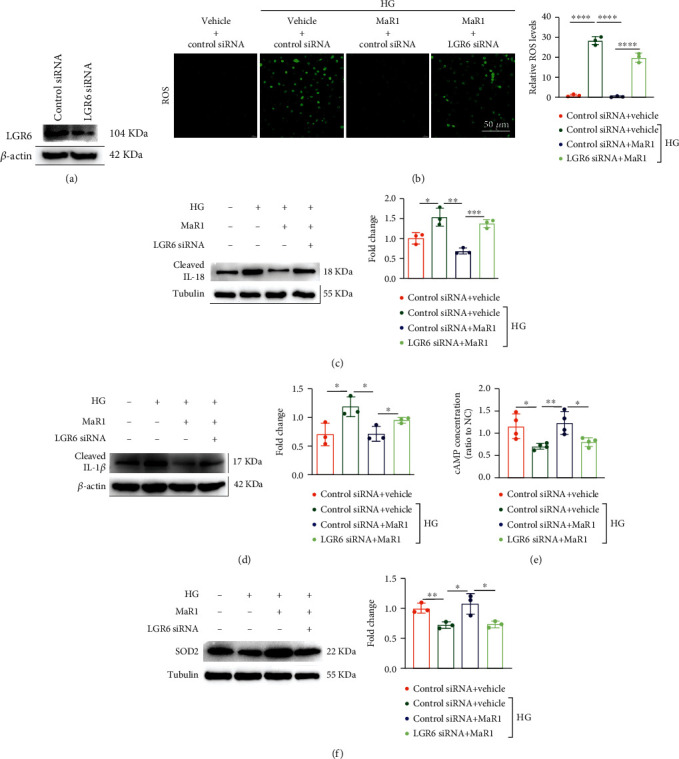
MaR1 alleviated high glucose-induced inflammation via LGR6-mediated antioxidant pathway. (a) Western blotting analysis images showing the knockout efficiency of LGR6 siRNA. (b) DCFH-DA probe was used to detect the levels of ROS in high glucose-stimulated HK-2 cells of LGR6 knock-down with MaR1 intervention. Bar: 50 *μ*m. (c) Western blotting analysis images showing the levels of IL-18 in cell culture supernatant of high glucose-stimulated HK-2 cells of LGR6 knock-down with MaR1 intervention. (d) Western blotting analysis images showing the levels of IL-1*β* in cell culture supernatant of high glucose-stimulated HK-2 cells of LGR6 knock-down with MaR1 intervention. (e) The levels of cAMP in high glucose-stimulated HK-2 cells of LGR6 knock-down with MaR1 intervention. (f) Representative western blotting analysis images showing the protein levels of SOD2 in high glucose-stimulated HK-2 cells of LGR6 knock-down with MaR1 intervention. All results are representative of three independent experiments. Data were expressed as mean ± SD; ∗P <0.05; ∗∗P <0.01; ∗∗∗P <0.001; ∗∗∗∗P <0.0001.

To further confirm that MaR1 alleviates DKD by LGR6 mediated antioxidant pathway, we next performed LGR6 knockdown in HK-2 cells (Figure 6(a)). Notably, LGR6 knockdown significantly blocked the anti-ROS effect of MaR1 on high glucose-treated HK-2 cells (Figure 6(b)). Similarly, LGR6 knockdown also blocked the anti-inflammation effect of MaR1 on high glucose-treated HK2 cells (Figure 6(c)–6(d)). Moreover, cAMP assay showed that the MaR1-induced increase of cAMP concentrations was completely inhibited by LGR6 knockdown in high glucose-treated HK-2 cells (Figure 6(e)). Consistently, up-regulated effect of MaR1 on SOD2 expression levels was also blocked in LGR6 siRNA transfected HK-2 cells (Figure 6(f)). These data indicate that MaR1 ameliorates high glucose-induced inflammation through LGR6-mediated cAMP/SOD2 antioxidant pathway.

## 4. Discussion

**Figure 7:**
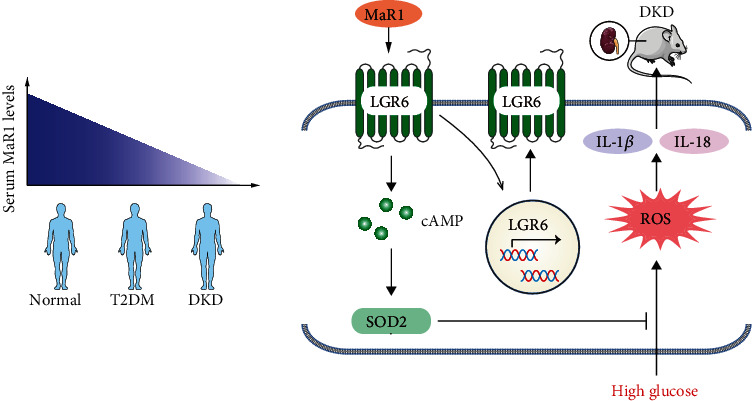
Schematic diagram depicting serum MaR1 levels in human and the mechanism by which MaR1 suppresses inflammation induced by high glucose via LGR6-mediated cAMP-SOD2 antioxidant pathway.

In our study, we identified MaR1 as a potential predictor and therapeutic target for DKD. Our data showed a strong negative correlation between MaR1 concentrations and the pathogenesis of DKD. Of note, MaR1 could alleviate the progression of DKD in vivo and reduce glucotoxicity-induced inflammation in vitro. Mechanistically, MaR1 up-regulated the expression of LGR6 and suppressed inflammation induced by chronic hyperglycemias via LGR6-mediated cAMP-SOD2 antioxidant pathway (Figure 7). Thus, the protective effect of MaR1 on DKD in our study lights a new way for DKD treatment.

Increasing evidences suggested that inflammation contributed to the pathogenesis and progression of DKD [49–51]. MaR1, synthesized from macrophages, is an extensive anti-inflammatory agent with conjugated triene double bonds, which plays a role in a variety of acute and chronic diseases [31, 52]. A correlation analysis study from Tian Miao et al. showed decreased levels of plasma MaR1 were associated with diabetic foot ulcer [53]. According to a recent study, urinary MaR1 were lower in the subjects with stage 3–4 nephropathy than in normal group and patients with stage 1 or 2 nephropathy [32]. Our research showed that serum MaR1 levels were significantly decreased in T2DM subjects with or without DKD compared with normal group, and were lowest in DKD patients among these 3 groups (Figure 1(a)). In addition, we also found that circulating MaR1 concentrations were negatively correlated with UACR (Figure 2(a)), and positively associated with eGFR (Figure 2(g)). Multivariate logistic regression analysis revealed that decreased serum MaR1 concentrations were the risk factor of T2DM without or with DKD (Supplemental Table 4). Thus, we speculate that circulating MaR1 levels may be a predictor of DKD. Further cohort studies are needed to clarify this.

Previous study [54] reported that MaR1 reduced liver TG content in ob/ob mice. Recently, Tian Miao et al. found that a significant negative relationship between TG and MaR1 levels [53]. Our results also showed a significant positive relationship between HDL-C and MaR1 levels (Figure 2(f)), supporting that MaR1 affect lipid metabolism. While after controlling for HDL-C, MaR1 remained statistically associated with both T2DM with or without DKD (Supplemental Table 4), suggesting that blood lipids might not be a mediator of this association. Animal research has reported MaR1 treatment was useful to reduce the hyperglycemia and the insulin resistance associated to obesity [55, 56]. Study in diabetic patients also showed a significant negative relation between MaR1 and insulin resistance [53]. Consistent with these, our cross-sectional study showed the serum MaR1 levels were significantly negatively correlated to HbA1c and duration of diabetes (Figures 2(b) and 2(c)). In addition, our study showed that MaR1 improved hyperglycemia in mouse model. According to studies in human and transgenic mice, most of insulin-dependent glucose transport is mediated by the insulin-dependent recruitment of the GLUT4 glucose transporter on the cell membrane of skeletal muscle and adipose tissue [57, 58]. Several studies have shown that activation of PI3K/Akt may be an essential requirement for insulin regulation of glucose uptake and GLUT4 in adipocytes [59] and skeletal muscle [60]. Therefore, MaR1 could promote glucose uptake by both adipose tissue and muscle through the activation of Akt and improve insulin resistance, contributing to lower blood glucose [55]. A longer duration of diabetes and poor glycemic control were associated with chronic complications of diabetes [61]. Therefor we speculate that MaR1 may be associated with chronic complications of diabetes, but further exploration is needed. Several studies have confirmed that MaR1 had the ability to control inflammatory disorders which was largely linked with its ability to inhibit neutrophil recruitment, reduce production of several proinflammatory cytokines and chemokines [62, 63]. Studies have revealed high NLR values might be a reliable predictive marker of early-stage DKD [64–66]. Our study found serum MaR1 concentrations were negatively correlated with neutrophil count and NLR (Figures 2(d) and 2(e)). Correlation analyses in this study showed that the decreased serum MaR1 concentrations were strongly associated with glucose and lipid metabolism disorders, renal dysfunction, and inflammatory cell activation, which supported circulating MaR1 levels might be a predictor of DKD.

Animal study by Yun Qiu et al. revealed MaR1 protected against renal ischemia/reperfusion injury (IRI) by inhibiting the TLR4/MAPK/NF-*κ*B pathways [67]. Cell study by Tang et al. showed that MaR1 inhibited NLRP3 inflammatory body and TGF-*β*1 and fibronectin in glomerular mesangial cells, thus speculating that MaR1 may protect DKD by reducing inflammation and early fibrosis [33]. In this study, we found that MaR1 treatment could reduce hyperglycemia and UACR as well as alleviate the progression of renal inflammation in DKD mice (Figure 3(b)–3(d)), which revealed MaR1 could protected against DKD.

Previous studies have shown that MaR1 can alleviate DKD by inhibiting ROS generation [33], but which upstream mechanism is unclear. Two distinct types of receptor molecules have been reported for MaR1, including LGR6 and ROR*α*. In our study, expression of LGR6 on DKD was weaker than that on normal group (Figure 4(c)). Subsequently, differences in LGR6 expression between the two groups were reconfirmed by immunohistochemistry or western blotting assay in human kidney (Figure 4(a)), mouse kidney (Figure 4(b)) and HK-2 cells (Figure 4(d)). Thus, we speculate that LGR6 plays an important role in the pathophysiological mechanism of DKD occurrence and development. LGR6, a GPCR, ectopic overexpression of which resulted in enhanced phagocytosis by the endogenous MaR1 [35]. In addition, LGR6 has been shown to play important roles in osteogenesis [68], the repair of wounds and hair regeneration [48], Remission of abdominal aortic aneurysm in murine [69]. Interestingly, our study found that MaR1 treatment increased LGR6 expression (Figure 4(e)–4(i)), suggesting that maybe there was positive feedback between MaR1 and LGR6.

Immunofluorescence of mouse kidney suggested that LGR6 were more expressed in renal tubules than in glomerulus in our study (Figure 4(c)). In addition, the expression of KIM-1, a marker of renal tubular injury, was significantly enhanced in DKD mice compared with normal mice (Figure 3(d)). As is known, proximal tubular epithelial cells have the reabsorption function and play a crucial role in the etiopathogenesis of DKD [70]. Thus, HK-2 cells were selected by us to explore the mechanism of MaR1 improving glucotoxicity. Activation of LGR6 as one of the MaR1 receptors has been shown to up-regulate cAMP expression [35]. Norihiro Sugino et al. demonstrated increased cAMP expression could up-regulate SOD2 [36]. As we all know, SOD2 played a key role in ROS suppression [37, 71]. Accumulating evidences have identified that hyperglycemia-induced oxidative stress is of particular interest in the development and progression of DKD [72, 73]. MaR1 as an anti-inflammatory mediator has been verified to inhibit the ROS production [74]. Consistent with previous studies, we found MaR1 treatment up-regulated SOD2 (Figure 5(e)–5(h)) by increasing cAMP (Figures 5(c) and 5(d)), and then inhibited ROS (Figure 5(a)) expression induced by hyperglycemias in mouse model and HK-2 cells. However, the above effects and the inhibitory effect on IL-18 and IL-1*β* of MaR1 were attenuated by siRNA knockdown of LGR6 in HK-2 cells (Figure 6), suggesting MaR1 alleviates DKD via LGR6-mediated cAMP-SOD2 antioxidant pathway.

## 5. Conclusion

Taken together, our results provide evidence for the proposed mechanism depicted in Figure 7. Our study demonstrates that decreased serum MaR1 levels were positively correlated with the development of DKD and MaR1 injection up-regulated the expression of its receptor, LGR6, and then alleviates DKD via LGR6-mediated cAMP-SOD2 antioxidant pathway. In a word, our present findings identify MaR1 as a predictor and a potential therapeutic target for treatment of DKD.

## Data Availability

The datasets used and analyzed in present study are available from the corresponding author.

## Supplementary Materials

Supplementary Materials Supplemental Table 1. List of primers used in the present study. Supplemental Table 2. Clinical and laboratory characteristics of the study participants. Supplemental Table 3. Spearman correlation coefficient of variables associated with circulating maresin 1 (MaR1) concentration in study population. Supplemental Table 4. Multivariate logistic regression about circulating MaR1 levels with the type 2 diabetes (T2DM) and diabetic kidney disease (DKD). Supplemental Figure 1. The average body weight of MaR1-treated DKD mice and vehicle treated DKD mice.
