# Supplementary material for: Maresin 1 Alleviates Diabetic Kidney Disease via LGR6-Mediated cAMP-SOD2-ROS Pathway
Source: Oxid Med Cell Longev. 2022 Apr 19;2022:7177889. doi: 10.1155/2022/7177889 (PMC9042615; doi:10.1155/2022/7177889)
Supplement: Supplementary Materials — Supplemental Table 1. List of primers used in the present study. Supplemental Table 2. Clinical and laboratory characteristics of the study participants. Supplemental Table 3. Spearman correlation coefficient of variables associated with circulating maresin 1 (MaR1) concentration in study population. Supplemental Table 4. Multivariate logistic regression about circulating MaR1 levels with the type 2 diabetes (T2DM) and diabetic kidney disease (DKD). Supplemental Figure 1. The average body weight of MaR1-treated DKD mice and vehicle treated DKD mice. [file 7177889.f1.docx]

**Supplementary Materials**

**Maresin 1 alleviates diabetic kidney disease via** **LGR6-mediated cAMP-SOD2-ROS pathway**

**Supplementary Data**

**Supplemental Table 1.** List of primers used in the present study.

**Supplemental Table 2.** Clinical and laboratory characteristics of the study participants.

**Supplemental Table 3.** Spearman correlation coefficient of variables associated with circulating maresin 1 (MaR1) concentration in study population.

**Supplemental Table 4.** Multivariate logistic regression about circulating MaR1 levels with the type 2 diabetes (T2DM) and diabetic kidney disease (DKD).

**Supplemental Fig. 1.** The average body weight of MaR1-treated DKD mice and vehicle treated DKD mice.

**Supplementary** **Table 1.** List of primers used in the present study.

| Species | NCBI Gene ID | Gene name | Sequence (5' to 3') | Length of product (bp) |
| --- | --- | --- | --- | --- |
| Mus | 329252 | LGR6 | F: GGTGTGGCTTTGAGTCCTCTGT  R: CCGAGGACTTTCATCCAGAGGA | 132 |
|  | 20656 | SOD2 | F: CAGACCTGCCTTACGACTATGG  R: CTCGGTGGCGTTGAGATTGTT | 113 |
|  | 14433 | GAPDH | F: AGGTCGGTGTGAACGGATTTG  R: TGTAGACCATGTAGTTGAGGTCA | 123 |
| Homo | 59352  6648  3606  3553 | LGR6  SOD2  IL-18  IL-1β | F: CAAGCTGGACCTGACAGACA  R: TGAAGAAGCTGGCACACATC  F: GCTCCGGTTTTGGGGTATCTG  R: GCGTTGATGTGAGGTTCCAG  F: GATAGCCAGCCTAGAGGTATGG  R: CCTTGATGTTATCAGGAGGATTCA  F: TTCGACACATGGGATAACGAGG  R: TTTTTGCTGTGAGTCCCGGAG | 194  92  121  84 |
|  | 2597 | GAPDH | F: CAATGACCCCTTCATTGACC  R: GACAAGCTTCCCGTTCTCAG | 52 |

**Supplemental Table 2.** Clinical and laboratory characteristics of the study participants.

|  | NC | T2DM | DKD |
| --- | --- | --- | --- |

| Sex (M/F) | 44(22/22) | 36(22/14） | 24(11/13) | |
| --- | --- | --- | --- | --- |
| Age (year) | 57.95±1.17 | 58.72±2.31 | 62.63±2.34 |  |
| BMI (kg/m^2^) | 23.11(21.17-25.75) | 24.70(22.93-26.69) | 24.08(21.42-26.14) |  |
| HbA1c (%) | 5.70(5.5-5.8) | 9.00(7.48-11.25)**^b^** | 10.10(8.38-11.8)**^b^** |  |
| Duration of diabetes (year) | _ | 4.50(1.00-10.00) | 11.50(8.00-18.00)^b^ |  |
| SBP (mmHg) | 123.89±2.41 | 129.22±3.33 | 135.88±3.92**^a^** |  |
| DBP (mmHg) | 74.82±1.69 | 75.19±1.88 | 78.04±1.51 |  |
| TC (mmol/L) | 4.30(2.78-4.97) | 4.14(3.51-4.97) | 4.66(3.78-5.25)**^ac^** |  |
| TG (mmol/L) | 1.84(1.16-2.78) | 1.43(0.93-2.16) | 1.63(1.08-2.24) |  |
| HDL-C (mmol/L) | 1.32(1.12-1.64) | 1.17(0.95-1.41)**^a^** | 1.11(0.96-1.34)**^a^** |  |
| LDL-C (mmol/L) | 2.86(2.31-3.59) | 2.43(1.86-3.02) | 2.98(2.44-3.59) |  |
| UA (mml/L） | 336.50(243.58-405.35) | 308.50(265.38-363.65) | 360.00(259.70-442.38) |  |
| Creatinine (umol/L) | 62.20(54.38-77.1) | 60.1(52.55-71.53) | 71.80(50.33-84.43) |  |
| eGFR (ml/min) | 105.00(99.00-114.00) | 99.7(89.85-109.7) | 87.6(62.25-107.03)**^b^** |  |
| UACR (mg/g) | 5.99(4.38-9.84) | 7.53(4.53-13.15) | 89.92(56.26-1241.01)**^bd^** |  |
| Neutrophil (×10^9^/L) | 3.39(2.69-4.21) | 3.83(3.39-4.65) | 4.37(4.07-5.45)**^b^** |  |
| Lymphocyte (×10^9^/L) | 1.84±0.08 | 1.57±0.06**^a^** | 1.76±0.13 |  |
| NLR | 1.93(1.51-2.42) | 2.69(2.22-2.87)**^b^** | 2.55(1.99-3.66)**^b^** |  |

| Data are presented as means ± SD for normally distributed values or the median (25-75th percentiles) for nonparametric values. NC: normal control; T2DM: type 2 diabetes without diabetic kidney disease; DKD: diabetic kidney disease; BMI: body mass index; HbA1c: hemoglobin A1c; SBP: systolic blood pressure; DBP: diastolic blood pressure; TC: total cholesterol; TG: triglyceride; HDL-C: high-density lipoprotein-cholesterol; LDL-C: low-density lipoprotein-cholesterol; UA: uric acid; eGFR: estimated glomerular filtration rate; UACR: urine albumin to creatinine ratio; NLR: neutrophil-lymphocyte ratio; ^a^ P< 0.05compared with NC; ^b^ P< 0.01 compared with NC;  ^c^ P< 0.05 compared with T2DM; ^d^ P< 0.01compared with T2DM. |
| --- |

**Supplemental Table 3.** Spearman correlation coefficient of variables associated with circulating maresin 1 (MaR1) concentration in study population.

|  | Serum maresin 1 | | Serum maresin 1 (age- and sex-adjusted) | |
| --- | --- | --- | --- | --- |
|  | r | P*-*value | r | P*-*value |
| Age (year) | -0.183 | 0.064 | — | — |
| Sex (M/F) | -0.036 | 0.717 | — | — |
| BMI (kg/m^2^) | -0.118 | 0.233 | -0.127 | 0.204 |
| SBP (mmHg) | -0.238 | **0.015** | -0.189 | 0.056 |
| DBP (mmHg) | -0.048 | 0.629 | -0.059 | 0.554 |
| TC (mmol/L) | -0.029 | 0.769 | -0.088 | 0.377 |
| TG (mmol/L) | -0.094 | 0.343 | -0.037 | 0.714 |
| HDL-C (mmol/L) | 0.326 | **0.001** | 0.353 | **<0.0001** |
| LDL-C (mmol/L) | 0.023 | 0.820 | 0.010 | 0.921 |
| HbA1c (%) | -0.622 | **<0.0001** | -0.500 | **<0.0001** |
| Duration of diabetes (year) | -0.738 | **<0.0001** | -0.512 | **<0.0001** |
| UACR | -0.518 | **<0.0001** | -0.203 | **0.041** |
| UA (mml/L） | -0.062 | 0.535 | -0.103 | 0.305 |
| Creatinine (umol/L) | -0.013 | 0.898 | -0.126 | 0.209 |
| eGFR (ml/min) | 0.249 | **0.011** | 0.222 | **0.025** |
| Neutrophil (×10^9^/L) | -0.360 | **<0.0001** | -0.342 | **<0.0001** |
| Lymphocyte (×10^9^/L) | 0.127 | 0.200 | 0.039 | 0.695 |
| NLR | -0.383 | **<0.0001** | -0.281 | **0.004** |

NC: normal control; T2DM: type 2 diabetes without diabetic kidney disease; DKD: diabetic kidney disease; BMI: body mass index; HbA1c: hemoglobin A1c; SBP: systolic blood pressure; DBP: diastolic blood pressure; TC: total cholesterol; TG: triglyceride; HDL-C: high-density lipoprotein-cholesterol; LDL-C: low-density lipoprotein-cholesterol; UA: uric acid; eGFR: estimated glomerular filtration rate; UACR: urine albumin to creatinine ratio; NLR: neutrophil-lymphocyte ratio.

| **Supplemental Table 4.** Multivariate logistic regression about circulating MaR1 levels with the type 2 diabetes (T2DM) and diabetic kidney disease (DKD). **(4a)** Circulating MaR1 levels with the T2DM. | | | | |
| --- | --- | --- | --- | --- |
| Models | Independent variable | Odds ratio (OR) | 95% CI | *P*-value |
| Model 1 | MaR1, pg/mL | 0.898 | 0.856 to 0.942 | **< 0.0001** |
| Model 2 | MaR1, pg/mL | 0.889 | 0.843 to 0.939 | **< 0.0001** |
| Model 3 | MaR1, pg/mL | 0.889 | 0.840 to 0.942 | **< 0.0001** |

Model 1: not adjusted, Model 2: adjusted for age, gender and BMI, Model 3: Model 2 plus blood pressure, and lipid profiles. The bold values indicate the *P*-values which were < 0.05. *CI:* confidence interval.

| **Supplemental Table 4.** Multivariate logistic regression about circulating MaR1 levels with the type 2 diabetes (T2DM) and diabetic kidney disease (DKD). **(4b)** Circulating MaR1 levels with the DKD. | | | | |
| --- | --- | --- | --- | --- |
| Models | Independent variable | Odds ratio (OR) | 95% CI | *P*-value |
| Model 1 | MaR1, pg/mL | 0.826 | 0.771 to 0.885 | **< 0.0001** |
| Model 2 | MaR1, pg/mL | 0.817 | 0.758 to 0.882 | **< 0.0001** |
| Model 3 | MaR1, pg/mL | 0.758 | 0.676 to 0.850 | **< 0.0001** |

Model 1: not adjusted, Model 2: adjusted for age, gender and BMI, Model 3: Model 2 plus blood pressure, and lipid profiles. The bold values indicate the *P*-values which were < 0.05. *CI:* confidence interval.

**Supplemental Fig. 1.** The average body weight of MaR1-treated DKD mice and vehicle treated DKD mice.


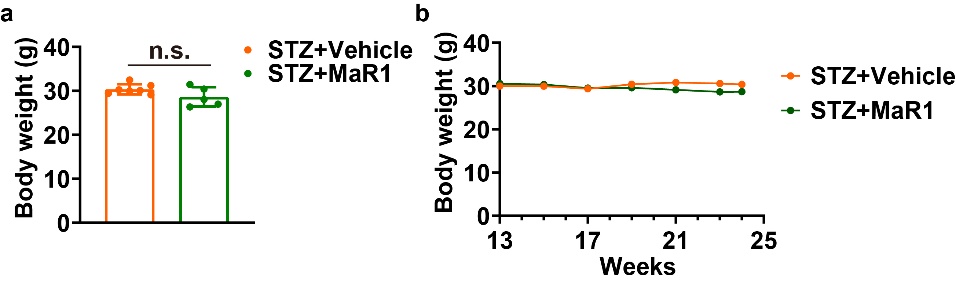


The average body weight of MaR1-treated DKD mice and vehicle treated DKD mice showed no significant difference. (a) Mean body weight of mice in indicated group before rats were sacrificed (24 weeks) (n=7 in DKD group and n=5 in DKD+ MaR1 group). (b) Mean body weight of mice in indicated group per week after MaR1 intervention (n=7 in DKD group and n=5 in DKD+ MaR1 group). n.s.: not significant.
